# Pragmatic cluster randomized trial to evaluate effectiveness and implementation of enhanced EHR-facilitated cancer symptom control (E2C2)

**DOI:** 10.1186/s13063-020-04335-w

**Published:** 2020-06-05

**Authors:** Lila J. Finney Rutten, Kathryn J. Ruddy, Linda L. Chlan, Joan M. Griffin, Jeph Herrin, Aaron L. Leppin, Deirdre R. Pachman, Jennifer L. Ridgeway, Parvez A. Rahman, Curtis B. Storlie, Patrick M. Wilson, Andrea L. Cheville

**Affiliations:** 1 Department of Health Sciences Research, Mayo Clinic, Rochester, MN USA; 2 Robert D. and Patricia E. Kern Center for the Science of Health Care Delivery, Mayo Clinic, Rochester, MN USA; 3 Division of Medical Oncology, Mayo Clinic, Rochester, MN USA; 4 Department of Nursing, Mayo Clinic, Rochester, MN USA; 5 Yale University School of Medicine, New Haven, CT USA; 6 Knowledge and Evaluation Research Unit, Mayo Clinic, Rochester, MN USA; 7 Center for Palliative Care, Mayo Clinic, Minnesota, USA; 8 Division of Community Palliative Medicine, Mayo Clinic, Rochester, MN USA

**Keywords:** Electronic health record, Neoplasm, Pain, Palliative care, Patient care team, Patient-reported outcome measure, Quality of life, Self-management, Systems integration, Survivor

## Abstract

**Background:**

The prevalence of inadequate symptom control among cancer patients is quite high despite the availability of definitive care guidelines and accurate and efficient assessment tools.

**Methods:**

We will conduct a hybrid type 2 stepped wedge pragmatic cluster randomized clinical trial to evaluate a guideline-informed enhanced, electronic health record (EHR)-facilitated cancer symptom control (E2C2) care model. Teams of clinicians at five hospitals that care for patients with various cancers will be randomly assigned in steps to the E2C2 intervention. The E2C2 intervention will have two levels of care: level 1 will offer low-touch, automated self-management support for patients reporting moderate sleep disturbance, pain, anxiety, depression, and energy deficit symptoms or limitations in physical function (or both). Level 2 will offer nurse-managed collaborative care for patients reporting more intense (severe) symptoms or functional limitations (or both). By surveying and interviewing clinical staff, we will also evaluate whether the use of a multifaceted, evidence-based implementation strategy to support adoption and use of the E2C2 technologies improves patient and clinical outcomes. Finally, we will conduct a mixed methods evaluation to identify disparities in the adoption and implementation of the E2C2 intervention among elderly and rural-dwelling patients with cancer.

**Discussion:**

The E2C2 intervention offers a pragmatic, scalable approach to delivering guideline-based symptom and function management for cancer patients. Since discrete EHR-imbedded algorithms drive defining aspects of the intervention, the approach can be efficiently disseminated and updated by specifying and modifying these centralized EHR algorithms.

**Trial registration:**

ClinicalTrials.gov, NCT03892967. Registered on 25 March 2019.

## Administrative information

Note: the numbers in curly brackets in this protocol refer to SPIRIT checklist item numbers. The order of the items has been modified to group similar items (see http://www.equator-network.org/reporting-guidelines/spirit-2013-statement-defining-standard-protocol-items-for-clinical-trials/).

**Table:** 

Title {1}	Pragmatic Cluster Randomized Trial to Evaluate Effectiveness and Implementation of Enhanced EHR-Facilitated Cancer Symptom Control (E2C2)
Trial registration {2a and 2b}.	Trial Registration: NCT03892967 (ClinicalTrials.gov) Protocol Version 1.0, June 3, 2019. Registered at clinicaltrials.gov.
Protocol version {3}	Protocol Version 1.0, June 3, 2019
Funding {4}	This research is supported by the National Cancer Institute of the National Institutes of Health (telephone 1–800–422-6237; award No. UM1 CA 233033).]
Author details {5a}	Department of Health Sciences Research (Drs Rutten, Griffin, and Storlie, and Mr. Wilson), Division of Medical Oncology (Dr Ruddy), Department of Nursing (Dr Chlan), Knowledge and Evaluation Research Unit (Dr Leppin), Division of Community Palliative Medicine (Drs Pachman and Cheville), and the Robert D. and Patricia E. Kern Center for the Science of Health Care Delivery (Drs Rutten, Griffin, Ridgeway, and Cheville, and Mr. Rahman), Mayo Clinic, Rochester, Minnesota, and Yale University School of Medicine (Dr Herrin), New Haven, Connecticut.
Name and contact information for the trial sponsor {5b}	This research is supported by the National Cancer Institute of the National Institutes of Health (telephone 1-800-422-6237; award No. UM1 CA 233033)
Role of sponsor {5c}	The content is solely the responsibility of the authors and does not necessarily represent the official views of the National Institutes of Health

## Introduction

### Background and rationale {6a}

Cancer and cancer therapy are associated with severe, disabling symptoms that have been causally linked to diminished survival, increased use of healthcare, reduced quality of life, unemployment, and non-adherence to recommended cancer treatments. The prevalence of inadequate management of symptoms has been reported to be as high as 90% [1], despite the availability of definitive evidence-based care guidelines and accurate and efficient assessment instruments, and is especially high among elderly patients and patients in rural areas. This disjuncture is needless because most symptoms can be mitigated [2, 3]. In response to this critical need, the National Cancer Institute (NCI) developed a funding opportunity announcement (No. RFA-CA-19-035) associated with the Beau Biden Cancer Moonshot Initiative to encourage research on implementation and evaluation of symptom monitoring and management systems for patients with cancer and survivors.

Patient-reported measures of symptom presence and severity are available, but meta-analyses and high-quality trials have failed to show that solely providing clinicians with such scores improves symptom management and associated outcomes [4–7]. Symptoms are challenging to manage. Successful management often requires diligent monitoring; multimodal care plans spanning behavioral, pharmacologic, and rehabilitative domains; and ongoing adjustment. These demands are particularly salient for the five most prevalent and destructive symptoms: sleep disturbance, pain, anxiety, depression, and energy deficit (fatigue) (SPADE). Emerging research demonstrates the substantial co-occurrence of cancer-related symptoms and, consequently, the artificiality of evaluating and treating symptoms in isolation [8–15]. Although guidelines exist for each symptom class, their enactment by disease-focused clinicians, who may lack both the training and the time necessary to coordinate symptom treatments, has proved infeasible. A more integrated approach would seek to manage commonly clustered symptoms (e.g. the SPADE pentad) in an integrated or sequenced manner [8, 10, 11, 13, 16–21].

Collaborative care approaches have proved difficult to implement at scale, yet recent advances in information technology permit their core components to be embedded in the generation of electronic health records (EHRs). This includes capabilities to leverage the EHR for remote and point-of-care electronic patient-reported outcomes (ePROs). EHR-driven approaches that include patient-care strategies with aligned symptom-specific goals, support for self-management training, and decision support for medication changes have considerable potential to improve the management of symptoms in patients with cancer [22–35]. Here we describe a randomized study to assess such an EHR-driven approach.

### Objectives {7}

Our institution is one of three research centers funded by the NCI as part of the Improving the Management of Symptoms During and Following Cancer Treatment (IMPACT) consortium [36]. The consortium consists of three research centers and a coordinating center that lends operational and scientific support to coordinate research activities across the consortium. We will evaluate a guideline-informed, enhanced, EHR-facilitated cancer symptom control (E2C2) system that uses two empirically supported levels of stepped care: level 1 provides low-touch, automated, self-management support for patients reporting moderate SPADE symptoms or limitations in physical function (or both). Level 2 provides nurse-managed collaborative care for patients reporting more intense (severe) symptoms or functional limitations (or both). We will conduct a hybrid type 2 stepped wedge pragmatic cluster randomized clinical trial among teams of clinicians who care for patients with solid tumors across all phases of cancer care in community care settings and clinics within an academic medical center [37]. Patients with hematologic malignancies will also be included in community practices.

The E2C2 intervention uses critical insights from the current evidence base to overcome barriers that have limited previous attempts to develop scalable, pragmatic approaches to symptom monitoring and management [4, 27, 38–53]. Specifically, E2C2 proposes to overcome historic challenges through three validated strategies that capitalize on increased EHR penetration and capabilities:

1. EHR-imbedded algorithms will deliver validated symptom-matched, self-management education at the point of symptom reporting for patients with moderate symptoms (level 1). This will enable registered nurse (RN) symptom care managers (SCMs) to focus their collaborative care efforts on patients with severe symptoms (level 2).
2. EHR clinical decision support tools that have been proved to improve the frequency of evidence-based care in other contexts [54, 55] will be used to enable oncologic clinicians to initiate needs-matched and validated symptom management approaches.
3. E2C2 will train and deploy designated RN SCMs in an EHR-based collaborative care environment. Cultivation of dedicated niche practitioners to achieve economies of scale has proved effective in both primary care and specialty care [56].

We will assess the impact of the E2C2 intervention on patient-reported symptoms and experience, use of the intervention, and experience of the care team over time through pursuit of the following aims:

#### Aim 1

To conduct a cluster randomized pragmatic trial with a stepped wedge design to test the hypothesis that a symptom-control–focused, collaborative-care–based E2C2 intervention will significantly reduce SPADE symptoms and improve physical function scores, reduce unplanned hospitalizations and visits to the emergency department (ED), improve adherence to cancer therapies, enhance quality of life, and extend survival.

#### Aim 2

To evaluate the hypothesis that use of a multifaceted, evidence-based implementation strategy to support adoption and use of the E2C2 technologies will improve patient and clinical outcomes.

#### Aim 3

To conduct a mixed methods evaluation to detect and identify disparities in the adoption and implementation of the E2C2 intervention among elderly and rural-dwelling patients with cancer, two groups that have had disproportionately more cancer symptoms and worse outcomes.

Successful completion of this trial will provide evidence for the impact of the E2C2 intervention on the management of SPADE symptoms and functional limitations and for various additional outcomes, including use of healthcare, adherence to cancer treatment, and survival.

### Trial design {8}

E2C2 will be evaluated through a pragmatic cluster randomized stepped wedge trial in which clinics and disease-specific teams are randomized in multiple steps. All patients receiving care for solid tumors in the Cancer Center at Mayo Clinic in Rochester, Minnesota (MCR) will be included in the study sample irrespective of type or stage of cancer. A similar sample will include patients receiving care from Mayo Clinic Health System (MCHS) community clinics in Minnesota and Wisconsin for solid tumors and for hematologic malignancies. The trial’s stepped wedge design randomizes the order of E2C2 implementation among 15 clusters. Clusters are defined at the level of the cancer-care team or clinic and are randomly assigned to one of five tranches to receive the implementation at staggered intervals. This design will allow data to be collected sequentially from all clusters for a minimum of 6 months of usual care before E2C2 and a minimum of 8 months during the implementation phase.

The intervention will begin according to assessments completed by patients for symptoms (SPADE) and physical function (ePRO). SPADE symptom scores from these assessments will be transformed into a graphic and narrative presentation for patients and clinicians that will include tools and clinical support for SPADE symptom management. To promote clinicians’ use of the EHR-based tools that support the intervention, we will apply validated implementation strategies, including practice alerts, audit and feedback, and practice facilitation.

The primary outcome for our trial is average SPADE symptom scores and physical function scores derived from patient-reported ePROs before medical oncology clinic appointments. The primary analysis will test whether any SPADE scores significantly improved after introduction of the intervention. Process measures will reflect stakeholders’ use of the EHR graphic and clinical decision support tools and will be abstracted from the EHR. In parallel with conduct of the E2C2 hybrid type 2 trial, mixed methods will be used to comprehensively assess engagement, system utility, and barriers and facilitators to implementation among key stakeholders and E2C2 users, including providers and patients. Specifically, we will assess the impact of implementation strategies (practice alerts, audit and feedback, and practice facilitation) on key study outcomes, including SPADE symptom scores and functional status. We will also use mixed methods to identify disparities in the adoption and implementation of the E2C2 intervention among elderly and rural-dwelling patients with cancer.

## Methods: participants, interventions, and outcomes

### Study setting {9}

**Fig. 1:**
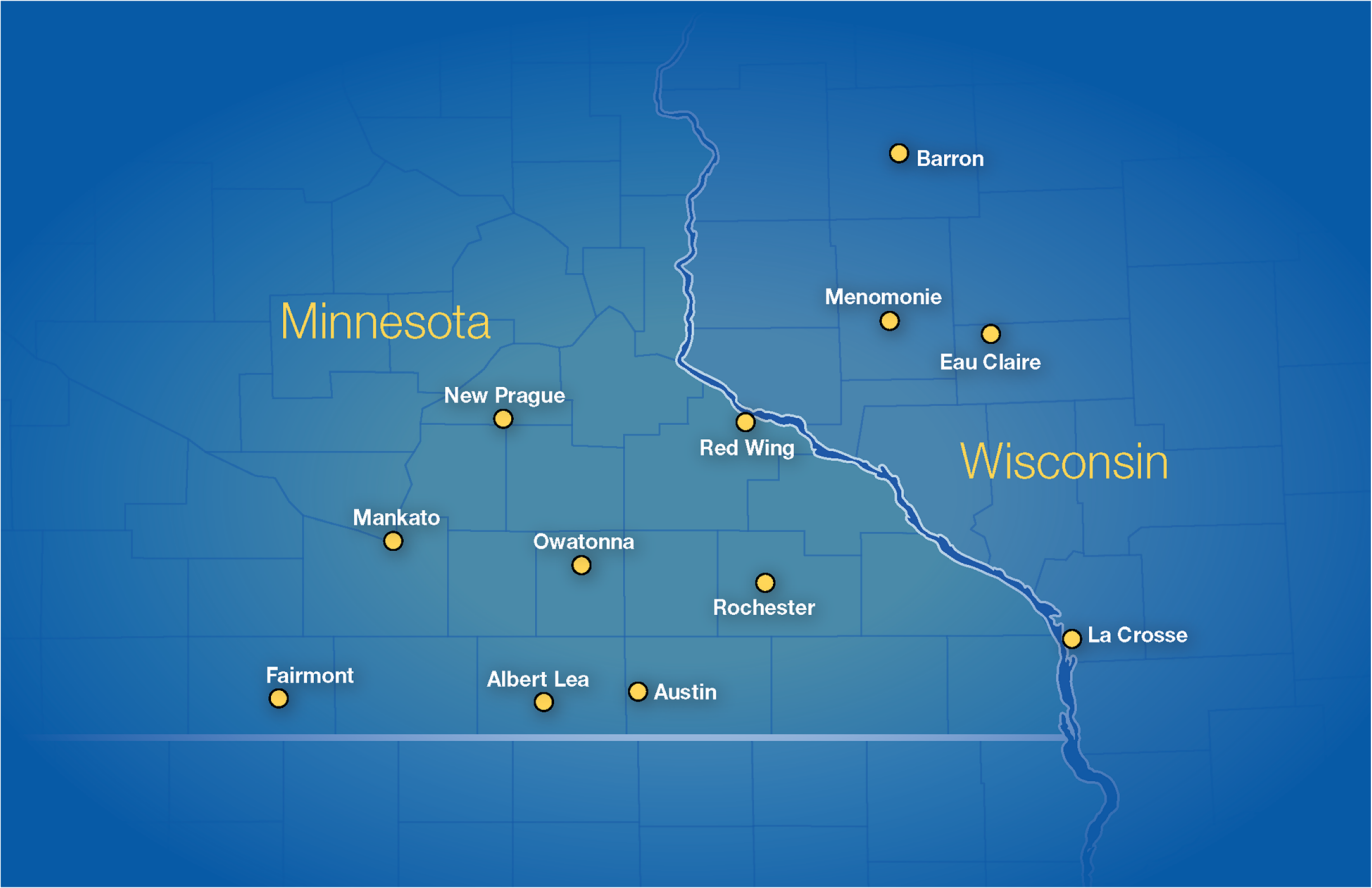
Locations of Participating Mayo Clinic Health System Sites in Minnesota and Wisconsin. (Used with permission of Mayo Foundation for Medical Education and Research)

The present study will be performed at MCHS sites in Minnesota and Wisconsin and at MCR. These practices provide cancer care to > 70,000 patients each year, 15,000 of whom have newly diagnosed cancers (approximately 10,000 of which are solid tumors) and hundreds of which are patients who have hematologic malignancy and receive care at MCHS sites, which serve 70 local communities in predominantly rural areas of Minnesota and Wisconsin. Figure 1 illustrates the locations of the participating sites; a complete list of sites is available from the corresponding author upon request.

MCR will be the primary research site. The patient populations served at MCHS sites tend to be older and more economically disadvantaged than those at MCR. Among the roughly 2500 patients who receive new cancer diagnoses annually and are treated in Mayo Clinic practices, 50% are rurally situated, 50% are elderly, and 18% are covered by Medicaid, which is comparable to the population of the upper Midwest [57]. The population’s ethnic and racial characteristics are also representative of the upper Midwest, with the exception that the population includes a higher percentage of Native Americans (about 3.8%). The expected distribution of types and stages of cancer in the study population parallels those described in the American Cancer Society 2018 summary [58], except that higher proportions of patients had melanoma, neuroendocrine tumors, and sarcomas, which reflect MCR subspecialties.

### Eligibility criteria {10}

This is a population-based study that will enroll all eligible adult patients served by clinicians in the Division of Medical Oncology at MCR and in hematology-oncology services at MCHS sites. This inclusive enrollment approach substantially increases the generalizability and external validity of our real-world pragmatic trial. Specifically, the E2C2 pragmatic trial will include all adult patients being treated or monitored for cancer or receiving survivorship care for cancer at the MCR medical oncology practice and the MCHS hematology-oncology practices. MCR patients in this trial will have solid tumors, and MCHS patients may have solid or liquid tumors. Data will not be used from patients who have indicated that they do not want their EHR data used for research according to the Minnesota Health Records Act [59].

### Who will take informed consent? {26a}

The Mayo Clinic Institutional Review Board (IRB) waived the requirement to obtain individual patient consent and deemed the effort a standard of care study.

### Additional consent provisions for collection and use of participant data and biological specimens {26b}

The protocol described herein was approved as a minimal risk study by the Mayo Clinic IRB on 1 November 2018 (IRB No. 18–007779). The IRB waived the requirement to obtain informed consent in accordance with 45 CFR 46.116 and waived Health Insurance Portability and Accountability Act (HIPAA) authorization in accordance with applicable HIPAA regulations. All changes to the protocol will be submitted to the IRB for review and approval. Persons who participate in the planned interviews will be asked to provide oral consent (see example in Additional file 1).

## Interventions

### Explanation for the choice of comparators {6b}

The stepped wedge design will allow us to compare outcomes at each clinic both across intervention groups and with historical controls at the same clinic, providing a rigorous assessment of the impact of E2C2 on managing SPADE symptoms among patients with cancer while also exploring factors relevant to its implementation across all sites.

### Intervention description {11a}

**Fig. 2:**
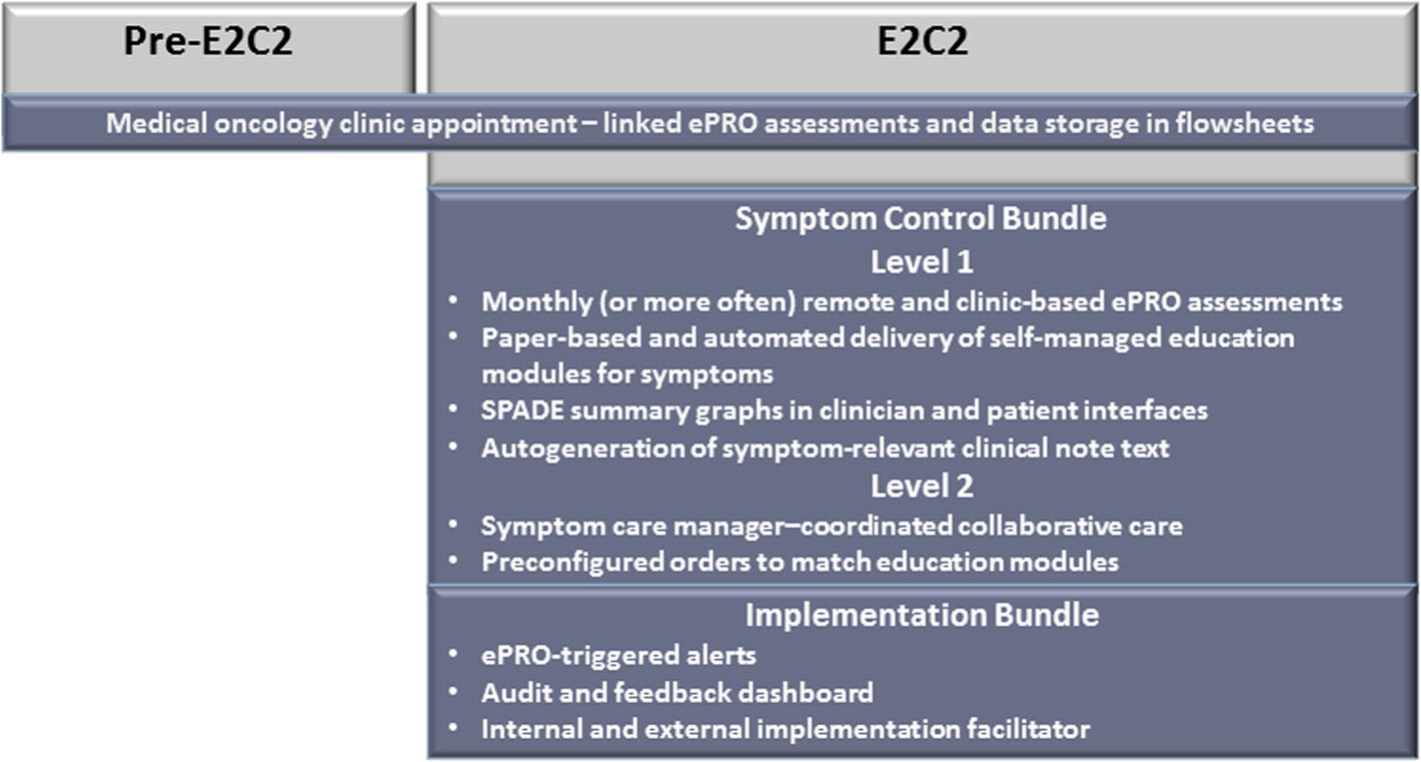
Intervention Overview. ePRO indicates electronic patient-reported outcome; E2C2, enhanced, electronic health record–facilitated cancer symptom control; SPADE, sleep disturbance, pain, anxiety, depression, and energy deficit

The intervention will consist of both a symptom control bundle and an implementation bundle, which will be introduced simultaneously. The E2C2 intervention will include patient- and clinician-directed elements designed to increase the frequency with which patients receive individualized, preference-concordant, and guideline-based care for their symptoms and to increase rates of symptom control. These bundles are summarized in Fig. 2 and described below.

#### Automated symptom monitoring

After the transition from pre-E2C2 to the E2C2 intervention, the ePROs used to assess SPADE symptoms and physical function will not change; however, assessment frequency will increase, particularly for patients reporting severe symptoms. After initiating E2C2, the symptom control bundle will be activated and clinicians will be able to access EHR clinical decision support tools for SPADE symptom management, including score tracking. Patients will be able to enter ePRO symptom and physical function assessment data through telephonic interactive voice response, web portal, smart phone, or tablet.

#### Symptom management bundle

##### Presentation of symptom data to patients and clinicians

To facilitate intuitive ePRO interpretation, dedicated views will be available in the clinician-accessible Synopsis section of the EHR [60]. The views will be specified according to the findings of Snyder et al. [60] to optimize accurate interpretation. Data collected on symptom intensity and context will be presented to clinicians in brief, autogenerated text that will populate their clinical notes with a patient’s longitudinal SPADE symptom scores and a hyperlink to the graphic Synopsis view.

##### Patient assignment to collaborative care level

Algorithms will be populated by automatically abstracted ePRO EHR data and used to determine the level of collaborative care that patients will receive. The triage algorithm will be based on the following: patient-reported SPADE symptom intensity; patient preference for RN SCM contact; and clinician referral. With the triage algorithm, patients will be initially assigned to one of three levels according to the severity of their symptoms: none or mild; moderate (level 1); or severe (level 2). Symptom intensity levels will be assessed with an 11-point numerical rating scale (NRS) and operationalized as follows: mild (0–3 points); moderate (4–6 points); or severe (7–10 points). Consideration was given to increasing the lower threshold for severe symptoms to 8; however, the decision was made to recognize that pain or depression rated 7 warranted RN SCM attention. Additionally, consideration was given to assigning patients with multiple, moderate symptoms to level 2 RN SCM. However, owing to ease of EHR specification and eventual dissemination, we ultimately chose to base the level assignment solely on intensity rather than number of symptoms. Patients may move between levels depending on treatment response and onset or worsening of symptoms.

##### Automated provision of guideline concordant, self-management education, and resource information to patients

All patients, irrespective of symptom level, will receive educational modules on SPADE symptom and physical function self-management. The modules will be paper booklets with site-specific inserts describing institutional, web-based, and community resources. The booklet will be mailed to all patients scheduled to see a specific provider after the provider’s cluster has begun the E2C2 intervention. At this appointment or subsequent appointments, if patients endorse moderate to severe SPADE symptoms or physical dysfunction, they will receive a portal-based link to videos and additional written content modules matched to any symptoms endorsed at a moderate or higher level. This digital content will be stored on a responsive website that can be accessed by phone, computer, or tablet. The web-based resource will be freely available to all patients in the intervention at any time. All module information is based on National Comprehensive Cancer Network guidelines, Mayo Clinic expert opinion, and the Oncology Nursing Society Putting Evidence Into Practice guides [61–63]. The content of the symptom modules will be cross-referenced annually against current guidelines. For each symptom, indications are provided for when and for what reasons the oncology team should be contacted.

##### Order preconfiguration for first-line symptom-directed medications to clinicians

The preconfigured medication or referral orders will be presented to clinicians caring for patients who are triaged to level 1 or 2. The order sets will be presented through Epic SmartSet (Epic Systems Corp Verona, Wisconsin) and will offer clinicians an efficient means of prescribing symptom-directed medications, specialist referrals, and allied health referrals that patients may request from the modules. Presenting preconfigured orders has been shown to have a small to moderate effect in improving patient outcomes [64, 65].

##### RN SCM–facilitated collaborative care (level 2 patients)

In addition to the interventions included for level 1 patients, the RN SCM will provide symptom-specific medication management, specialist referrals, integrative health approaches, and reinforced self-management for patients who express that they would like assistance with severe symptoms or functional impairment. Additionally, if providers in activated clusters believe that their patients with moderate symptoms may benefit from RN SCM care, they can advance patients to symptom level 2. RN SCM involvement is designed to optimally align treatment with each patient’s specific symptoms, preferences, and responses to therapy or interventions; thus, the frequency and content of nurse calls will vary with our treat-to-target approach. We will systematically document in detail the frequency, duration, and content of all RN SCM telephone calls. This will allow us to secondarily examine the independent effect of the intensity and content of RN SCM contacts as mediators of outcomes.

The RN SCM will contact patients in response to ePRO symptom-triggered alerts and trend reports. The RN SCMs will monitor trend reports weekly, respond to automated monitoring clinical alerts and patient calls daily, configure orders for the oncology care team through the EHR, and serve as the coordinator between patients and the oncology care team. For E2C2, the RN SCMs will have weekly case management sessions with the palliative care physician and additional clinical specialists as needed to review new level 2 patients and any patients not responding to therapy or suggested interventions.

### Criteria for discontinuing or modifying allocated interventions {11b}

Although interventions will be allocated at the site level and introduced to individual patients as clinical care, patients always have the option of refusing care or declining participation in certain aspects of care.

### Strategies to improve adherence to interventions {11c}

We will support uptake of E2C2 with an implementation bundle, a multifaceted strategy to encourage clinicians’ adoption of the EHR-based clinical decision support tools that combines three evidence-based implementation strategies: practice facilitation; point-of-care best practice prompts; and audit and feedback [66]. For practice facilitation, we will recruit clinical champions (physicians, nurse practitioners, or physician assistants) from each cluster and train them to support adoption and use of the E2C2 EHR clinical decision support tools; we will also familiarize clinicians with the RN SCM role [66–68]. The facilitator will also provide hands-on training and support to clinicians to foster understanding of and engagement with the symptom monitoring and management system [66–68]. Point-of-care computer alerts will be embedded within the EHR as Epic Best Practice Advisories, which will appear as alerts on the clinician EHR interface [69, 70]. Audit and feedback clinical performance data will be collected through automated Epic EHR tracking [70]. Clinical champions will be responsible for developing their own cluster-level engagement strategies and, with support from the research team, reporting on the educational and engagement strategies they deploy. Additionally, they will provide feedback to the research team on the utility and function of the algorithms.

### Relevant concomitant care permitted or prohibited during the trial {11d}

The trial is being conducted as an embedded pragmatic trial and will therefore occur within the context of routine care delivery.

### Provisions for post-trial care {30}

No special provisions for ancillary or post-trial care or compensation for harm were developed since the trial is an embedded pragmatic trial occurring within the context of routine care delivery.

### Outcomes {12}

#### Primary outcomes

NRS scores used to assess SPADE symptoms and physical function will serve as both the E2C2 primary outcome and the critical component of the E2C2 intervention since NRS scores populate the EHR algorithms that determine whether patients are triaged to level 1 or level 2, and whether patients are offered specific intervention components (e.g. education or medications).

#### Secondary outcomes

In addition to our primary outcomes, we will assess secondary outcomes including depression, anxiety, pain, and additional measures of physical function. We will also assess use of healthcare, including hospitalizations, visits to the ED, visits to the outpatient clinic , and calls to the oncology care team. Vital status will also be assessed.

### Participant timeline {13}

**Fig. 3:**
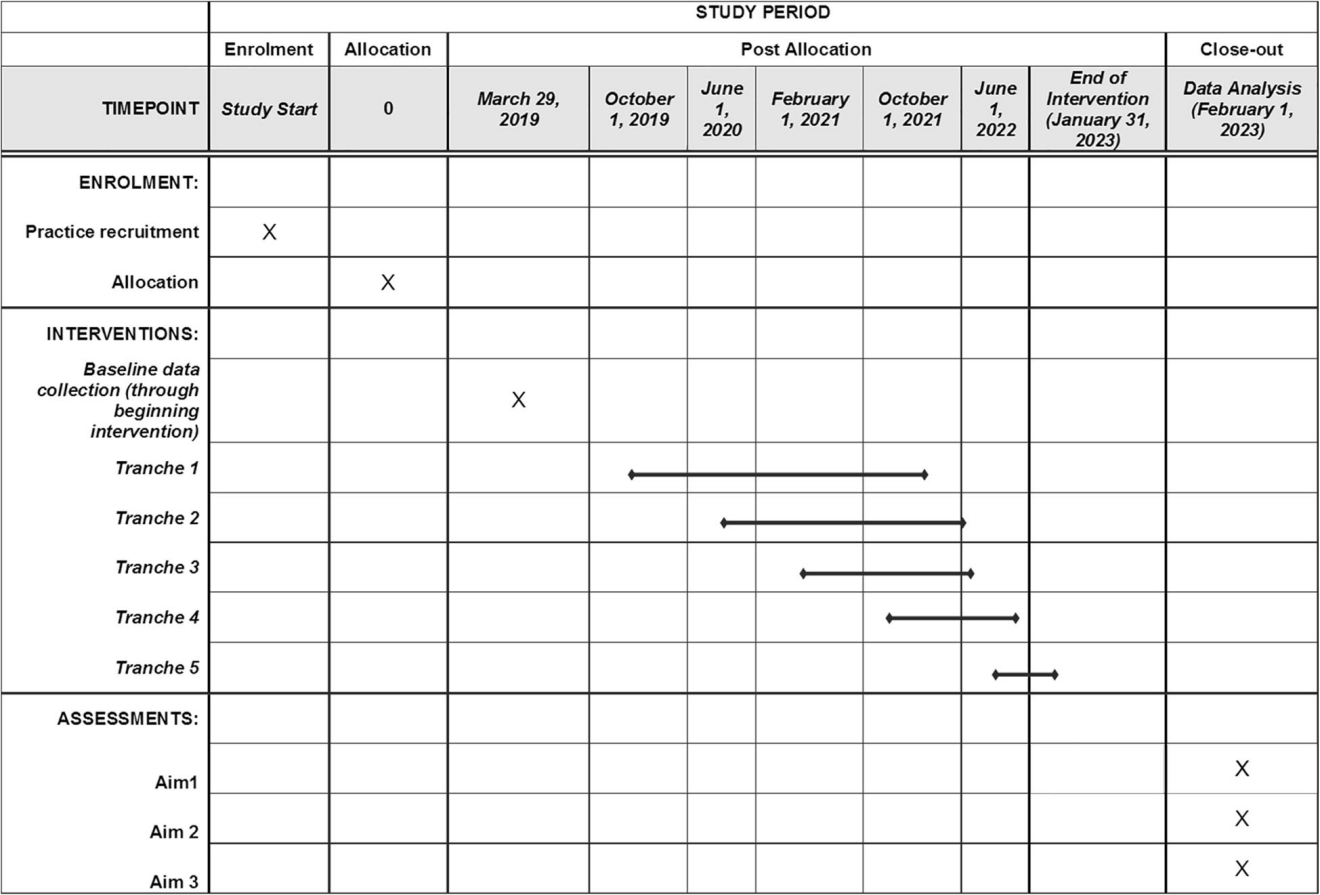
SPIRIT Diagram

The timeline for this effort is illustrated, in part, in Fig. 3, which shows the timeline for each step in our trial. The overall effort is funded as a 5-year project with 6 months of planning and preparation time at the start of the 5-year period and 6 months for analysis and dissemination at the end of the 5-year period.

### Sample size {14}

Stepped wedge cluster randomized trials typically have more statistical power than other cluster randomized designs [71] because each cluster can serve as its own control, accounting directly for the within-cluster correlation of outcomes. Because of the complex nature of the design and statistical model, we will estimate statistical power with simulation [72].

The sample size will be determined by the number of patient visits to the disease groups in the oncology practice at MCR and in the clinical practice sites included in the surrounding community practices in the MCHS as well as by the number of clusters included in our trial (15 clusters). As noted above, we estimate 40,000 newly diagnosed cancers over the study period. Since the present study also includes patients with existing tumors diagnosed in the past, if the patients are still receiving care from a medical oncologist at MCR or a hematologist-oncologist at MCHS, we expect the true sample size to be much larger than 40,000. According to NRS responses to pain and fatigue assessments that are currently imbedded in the MCR medical oncology clinic intake work flow, we expect that roughly two-thirds of patients will report at least one moderately intense symptom during treatment.

**Fig. 4:**
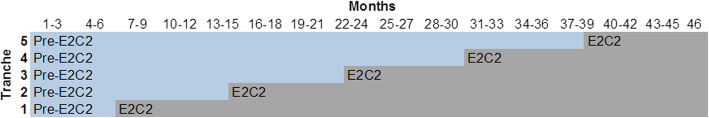
Stepped Wedge Cluster Design for the Enhanced, Electronic Health Record–Facilitated Cancer Symptom Control (E2C2) Trial

**Fig. 5:**
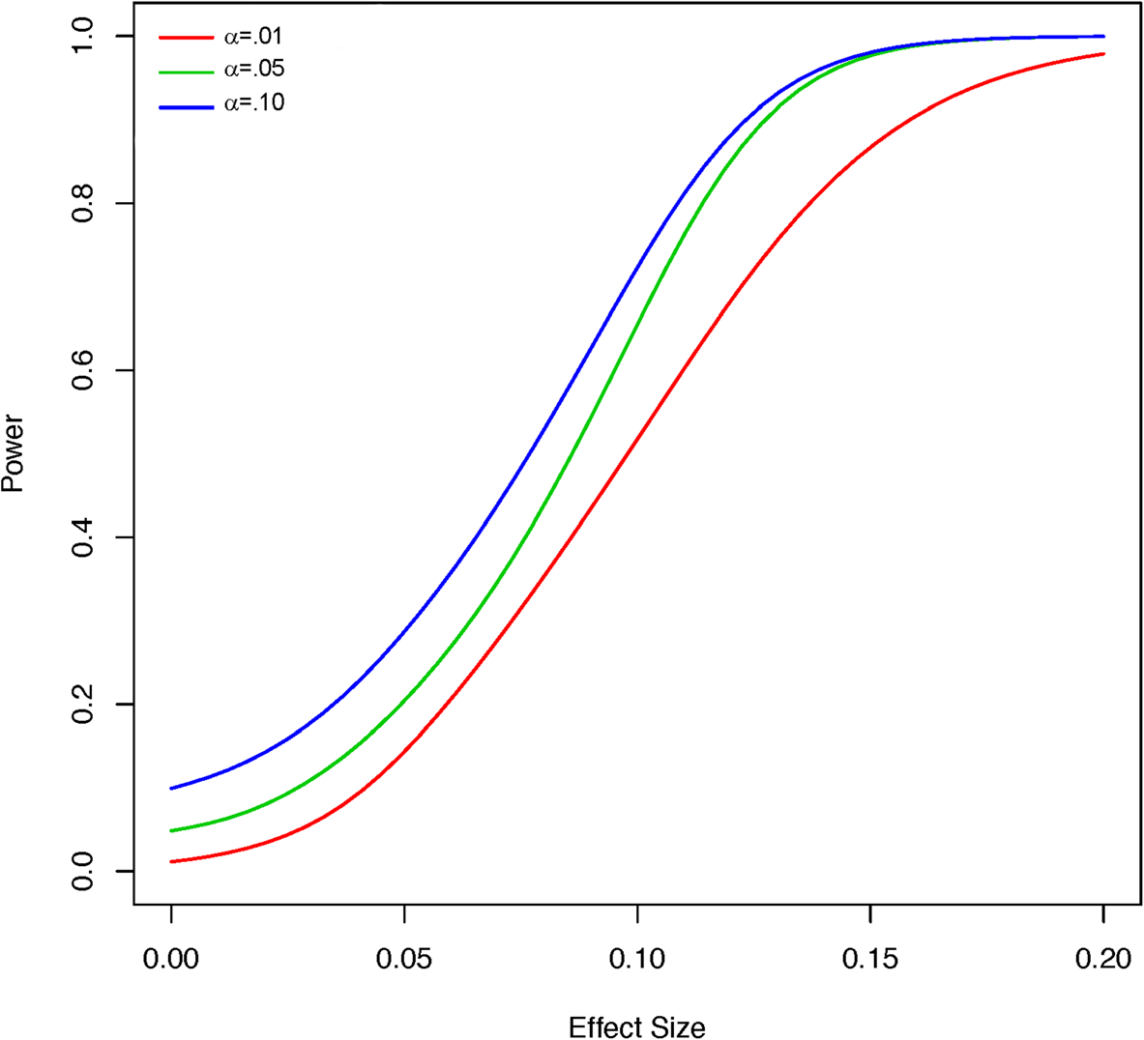
Simulated Power in Relation to Effect Size

From these conservative estimates, a version of the statistical model above was fit to 79 weeks of preliminary baseline data (i.e. with no intervention effect *X*_*kt*_) to estimate the parameters (e.g. ***ν***_*A*_, ***Σ***_*A*_) needed to simulate longitudinal data for the 15 clusters. Clusters were randomly allocated to the five steps in Fig. 4, and treatment effects due to intervention were generated on a grid of eight values *ν*_1*B*_ = 0.00, 0.01, 0.02, 0.03, 0.05, 0.10, 0.15, 0.20, with the remaining *ν*_2*B*_ = *ν*_3*B*_ = *ν*_4*B*_ = *ν*_5*B*_ = 0 to assume the most conservative case (i.e. only a single symptom is affected by the intervention). The cluster effects were then generated according to ${\boldsymbol{B}}_k\sim N\left({\boldsymbol{\nu}}_B,\sqrt{\nu_{1B}}{\boldsymbol{\varSigma}}_A\right)$. This data generation process was used to simulate 100 datasets under each of the eight grid values for *ν*_1*B*_. The full model above was then used to estimate ***ν***_*B*_ and test the hypothesis that all *ν*_*jB*_ = 0, through simultaneous credible bounds [73]. The results of the simulation are provided in Fig. 5. As can be seen in Fig. 5, the test results are close to the nominal level (i.e. α = 0.01, α = 0.05, or α = 0.10) when there is no effect. However, the power increases quickly for detection of a small average score difference of 0.10 with 70% power (at α = 0.05) and a difference of 0.20 with nearly 100% power.

For evaluation of the implementation bundle, we will summarize patient characteristics (sex and age) by intervention status (baseline and intervention). We will also summarize all survey responses by cluster implementation status, including clinician characteristics (age, sex, and years of practice). Analyses will be based on intention to treat at the patient level and at the cluster level [74]. All methods will account for correlation of outcomes across patients and within clusters.

We will also assess the effect of process measures on SPADE scores and other clinical outcomes. To assess the effect of clinician experience on implementation processes, we will assess the relationship between clinician scores and implementation outcomes by modeling each outcome as dependent on clinician scores. For count data, we will use appropriate models and select standard or zero-inflated Poisson or negative binomial models according to the Akaike information criterion. For binary measures, we will use logit models. For each outcome we will estimate bivariate models, including only one survey score, a model with all scores, and a final model that also includes clinician characteristics.

To evaluate the impact of the implementation bundle on patient outcomes, we will use similar assumptions as in Aim 1 to estimate the power to detect a meaningful difference in SPADE symptoms. To evaluate the impact of the intervention on clinician experience scores, according to prior response rates, we assume that 104 (95%) clinicians will complete surveys during both time periods; thus, the study will have 80% power to detect a change in scores of 0.27 standard deviations, a standardized effect that is conventionally considered small [75].

### Recruitment {15}

Our estimated sample size and power estimates were based on actual patient volumes to ensure the feasibility of planned enrollment. It is presumed that patient volumes will remain relatively stable over the enrollment period. Our design has five steps with three clusters randomized to the intervention at each tranche (Fig. 4). Each step will begin with a baseline assessment period (pre-E2C2), and initiation of the E2C2 intervention in each tranche will occur 8 months apart. During the pre-E2C2 before the first step, no intervention will be implemented; data collected during this period will provide a within-site control group for each cluster.

## Assignment of interventions: allocation

The SPIRIT diagram is shown in Fig. 3. The diagram illustrates the allocation of physician clusters into steps for a cluster randomized trial with a stepped wedge design. Clinical team members included in the study will be grouped into clusters. In the MCR practice, each cluster will be for a type of cancer (e.g. breast or colorectal cancer). In MCHS practices, each cluster will include site-specific oncology providers. These clusters will be the units for randomization for the intervention.

### Sequence generation {16a}

Random assignment was stratified by site (MCR or MCHS), patient volume, and, for teams caring for the same cancers, type of cancer. As a proxy for total patient volume, we estimated from pilot data in our cancer registry that 10,000 patients with newly diagnosed cancer would provide data in each of the 4 years of the grant cycle (a total of 40,000 patients). Counting annual visits for these patients in the most recent year, we stratified clusters into three groups: < 4000 visits annually; 4000–8000 visits annually; and > 8000 visits annually.

### Concealment mechanism {16b}

Clusters were allocated for all five steps in a single randomization generated by our lead statistician. Only the principal investigator and the lead statistician have knowledge of the randomization plan. Leadership at each allocated site will be alerted to the randomization several months before the start date. During the month before the intervention start date, randomly assigned care teams will be informed of the intervention and provided information and training.

### Implementation {16c}

The plan for implementation of allocation is illustrated in the SPIRIT diagram (Fig. 3) and in Fig. 4. To balance cluster characteristics, we stratified randomization on cluster size, type of cancer, and site.

## Assignment of interventions: blinding

### Who will be blinded {17a}

Since the intervention is occurring as part of clinical care and patients are therefore not asked to consent to participate in research, they will remain blinded to their participation in a research study.

### Procedure for unblinding if needed {17b}

No circumstances have been delineated under which unblinding would occur, nor have procedures for doing so been developed because the intervention will occur in this embedded pragmatic trial as clinical care.

## Data collection and management

### Plans for assessment and collection of outcomes {18a}

We will use multiple data sources for the E2C2 study analyses and identify patients and clinicians for qualitative data collection. The majority of data will be electronically abstracted either directly from the Epic EHR or from the Mayo Clinic data warehouse, the Unified Data Platform, which includes Epic and other legacy EHR and departmental systems and administrative billing data.

#### Demographic, clinical, clinician, and site data

Patient characteristics abstracted from the EHR will include sociodemographic and clinical variables. Potentially time-varying characteristics (e.g. regional and systemic cancer treatments) will be abstracted at monthly intervals. Validated natural language processing algorithms will be used to ascertain unstructured data elements [76, 77]. Clinician characteristics will be captured from departmental records, publicly accessible curricula vitae, and the EHR. Site-level data will include setting (community hospital, free-standing clinic, multispecialty group, or primary care clinic), available on-site cancer treatment services (e.g. surgical and radiation oncology), available on-site supportive care (e.g. pain management and psychology) and allied health services (e.g. physical therapy), location, and annual patient volume.

#### Primary outcomes

##### SPADE symptoms and physical function

Patients will complete NRSs for SPADE symptoms and physical function before medical oncology clinic appointments at all stages of the trial (i.e. before and after E2C2 initiation). ePROs will be more frequently administered to patients cared for by clinicians in activated E2C2 clusters, outside their appointment-linked assessments (at least monthly) and to symptomatic patients as part of the intervention. To avoid potential bias from oversampling of symptomatic patients, only SPADE symptom NRSs collected in association with clinic appointments will be included in the primary analysis.

Simple 11-point NRSs have been used for decades in the assessment of diverse latent traits among patients with cancer of all types and stages [78–80]. These single-item assessments have been the measures used most often for quality of life and symptoms in NCI cancer control clinical trials [81]; they feature prominently in the most reliable and valid symptom assessment tools, both generic and cancer-specific [82, 83]. The NRS used in E2C2 to assess the SPADE symptoms and physical function will be patterned on the extensively validated MD Anderson Symptom Inventory and the Edmonton Symptom Assessment System for the 24-h recall period and the verbal anchors (e.g. from no pain to worst possible pain) [84, 85], NRSs have been shown to be more responsive than either Likert or verbal rating scales [86] and to be preferred by patients over visual analogue scales [87].

#### Secondary outcomes

##### Patient-Reported Outcomes Measurement Information System computer adaptive tests

Depression, anxiety, pain, and physical function will be assessed with the Patient-Reported Outcomes Measurement Information System (PROMIS) computer adaptive tests (CATs) (http://www.healthmeasures.net/explore-measurement-systems/promis) up to every 4 months (before medical oncology or hematology-oncology visits either at the visit with a tablet or before the visit through the patient portal). In addition to strong endorsement by the National Institutes of Health [88, 89], use of CATs is justified because item response theory (IRT)-based instruments generally have better discrimination across the entire trait range than legacy patient-reported outcomes because they are less prone to floor and ceiling effects [90, 91]. Moreover, administering IRT-modeled banks with CATs enhances the efficiency and precision of measurement compared with short forms [92, 93]. The PROMIS item banks used in E2C2 have been validated, with robust IRT calibration across different clinical populations [94–96]. Each of the research centers participating in IMPACT is planning to use the PROMIS CATs to facilitate data harmonization and comparisons across practice settings.

##### Use of healthcare

Use of healthcare for E2C2 will consider healthcare encounters, including hospitalizations, visits to the ED, visits to the outpatient clinic, and calls to the oncology care team. EHR entries and administrative billing data will be aggregated to construct a comprehensive dataset of all clinical encounters. Data collected for hospitalizations will include admission and discharge diagnoses, length of stay, admission or transfer to the intensive care unit, and whether admission was planned for cancer treatment or unplanned (and if unplanned, whether admission was initiated from the ED or an office visit). For ED encounters, we will record diagnoses and whether an encounter resulted in admission to hospital or the intensive care unit. Clinic visits will be identified from billing data, which will include *Current Procedural Terminology* codes, *International Classification of Diseases, Tenth Revision* codes, location, and clinician National Provider Identifier (NPI) numbers. We will be able to ascertain clinician discipline and specialty from the NPI numbers. Calls to the oncology care team are not billed but are reliably captured in the EHR. A recent audit established that 87% of calls to medical oncology practitioners were recorded in the EHR and that rates of capture did not vary systematically across physicians and mid-level providers. However, call characteristics (e.g. duration) were not reliably recorded. Therefore, we will use count data in analyses to model call frequency.

The majority of patients’ use of healthcare will occur at Mayo Clinic facilities. Established patterns suggest that 75%–80% of patients’ care will occur at a Mayo Clinic facility and that this frequency will not vary across E2C2 clusters or during phases of the E2C2 intervention (e.g. pre-E2C2). However, we will use imputation methods and sensitivity analyses to assess the impact of mis-ascertainment of utilization data.

##### Vital status

Vital status will be verified through death certificates, the Mayo Clinic EHR, next-of-kin reports, the Mayo Clinic Tumor Registry, and the Social Security Death Index website.

#### Implementation evaluation and outcomes

##### Context assessment

We will use a 12-item Acceptability of Intervention Measure (AIM), Intervention Appropriateness Measure (IAM), and Feasibility of Intervention Measure (FIM) to assess system-level context during the time frame before implementation. Approximately 20 system-level stakeholders (e.g. administrators, practice leadership, and information technology specialists) will be recruited during the pre-E2C2 stage to complete the AIM-IAM-FIM survey. System-level stakeholders will be invited to complete an individual interview at the time of their pre-E2C2 survey. Interviews will comprise 15- to 30-min elaborations of the same AIM-IAM-FIM constructs, with prompts informed by the Consolidated Framework of Implementation Research [97].

Immediately before the E2C2 intervention is begun, all care team members within a given cluster will be invited to complete the same 12-item AIM-IAM-FIM survey. Participants will be recruited at cluster engagement kick-off meetings and data will be used to ascertain cluster-level readiness for implementation. The aim of this survey is to assess implementation context; the aim of the data collection is to understand and track implementation context. The survey will be re-administered 2–4 months after the intervention is initiated and 12 months after that assessment.

##### Process assessment

To characterize the work for stakeholders to implement E2C2, cluster care team members will also complete a 23-item NoMAD survey immediately before intervention is initiated, 4 months after intervention initiation, and 12 months after that assessment. The NoMAD survey, based on normalization process theory [98], includes assessment of users’ perceptions of intervention leadership, integrated workflow, training adequacy, organizational understanding, perceptions of the value of the intervention, and its impact on working relationships. The NoMAD instrument is treated as an ordinal scale and an average score within each construct can be calculated for individuals and clusters.

Care teams will also be invited to participate in focus groups in coordination with scheduled site visits and survey assessments (two focus groups per care team cluster). Focus groups will function like reflective team meetings and will be recorded with permission and transcribed for analysis. Additional interviews and focus groups may also be scheduled with key stakeholders and care team members to explore insights from the survey findings. Finally, implementation documents, including executive team meeting minutes, project correspondence and materials, and study logs maintained by study team members will be collected for textual analysis of how the intervention unfolded in practice.

##### Patient ePRO use

We will follow a mixed-methods sequential explanatory design to evaluate patient variation in the implementation of the intervention among rurally based, elderly, and rurally based elderly patients. Our evaluation will be guided by the Reach, Effectiveness, Adoption, Implementation, and Maintenance (RE-AIM) framework [99, 100]. We will evaluate the following:

1. Reach—the intervention is accessed across age groups and patients in rural and urban areas
2. Effectiveness—whether the intervention reduces disparities in symptom control among the elderly and those living in rural areas
3. Implementation and maintenance—whether the intervention and its components are implemented consistently with these groups

For each step, we will extract data from the EHR to assess quantitative outcomes (response rates and changes in SPADE symptom scores) and follow analysis of the quantitative data with qualitative inquiries to help explain and elaborate on the quantitative findings.

### Plans to promote participant retention and complete follow-up {18b}

#### Adoption, fidelity, and penetration

Measures of actual adoption, fidelity, and penetration will be abstracted from administrative data and utilization tracking data mined with the EHR. Process measures reflecting patients’ and clinicians’ use of the E2C2 EHR tools will be captured automatically by the EHR. Process measures for patients will include the following:

1. Frequency and duration of accessing the SPADE symptom self-management educational modules through the web portal
2. Frequency of requesting print versions of the modules
3. Frequency of accessing the designated SPADE symptom graphs through the portal

Process measures for clinicians will include the following:

1. Retention of autogenerated text in clinical notes
2. Frequency of accessing the SPADE Synopsis view
3. Frequency of issuing preconfigured orders for SPADE treatments
4. Frequency of issuing orders configured by the level 2 RN SCMs
5. Clinician responses to Best Practice Advisory alerts
6. Frequency of clinician links from the alerts to the SPADE symptom Synopsis view and the preconfigured SmartSet orders
7. Duration and frequency with which clinicians view the audit and feedback dashboard
8. Frequency with which clinicians use the dashboard to gain further information about their performance

Individual and group quarterly performance data will be provided to clinicians as feedback through the dashboard. Upon initiation of the E2C2 intervention, individual performance data will be collected and a dashboard will be presented weekly on the clinician’s landing page at first login to the EHR. The EHR will record the frequency, duration, and detail of dashboard access and use. These data will be used to assess the impact of audit and feedback on outcomes.

#### Patient interviews

We will complete individual interviews with patients in E2C2—especially those who live in rural areas or are elderly—to understand their experience with E2C2 and symptom management. We will use a stratified, purposeful sample of patients or proxies for interviews. Starting with the first clusters randomly assigned to the E2C2 intervention, we will identify patients who are aged < 65 years and living in rural areas, as defined by the 2013 US Department of Agriculture’s Rural-Urban Continuum Codes [101], and patients aged ≥ 65 years who live in urban and rural areas. We will then identify high and low ePRO reporters during the first 3 months of the intervention and recruit from both groups for interviews to be conducted in months 4–6. Our second set of interviews will be conducted during months 18 and 19 and will assess barriers and facilitators to symptom control and the effectiveness of the E2C2 intervention in promoting symptom control. The semi-structured interview guide will be based on domains consistent with the RE-AIM framework. The interview schedule will follow an iterative process of data collection and analysis to ensure the identification and testing of analytic categories.

### Data management {19}

All patient and medical records data are securely stored behind an electronic firewall. Patient and medical records data will not be released outside the institution without specific data-sharing agreements. Additional study data will be stored on separate, password-protected, secure servers; only study personnel will have access to these data. All results will be reported in aggregate; no individual-level patient or provider data will be identifiable in study reports.

### Confidentiality {27}

Confidentiality and security of patient data will be ensured through adherence to institutional policies and procedures for secure storage and maintenance of medical records data. Patients will participate in the study only through their engagement in routine care; thus, the institutional clinical policies and procedures for maintaining patient privacy and confidentiality with respect to communications, paperwork, and EHR documentation and access will ensure patient privacy.

### Plans for collection, laboratory evaluation, and storage of biological specimens for genetic or molecular analysis in this trial/future use {33}

Plans for collection, laboratory evaluation, and storage of biological specimens for genetic or molecular analysis are not applicable to the current trial.

## Statistical methods

### Statistical methods for primary and secondary outcomes {20a}

We will summarize patient characteristics (e.g. sex and age) by intervention status (pre-E2C2 or E2C2). Analyses will be based on intention to treat at the patient level and at the cluster level [74]. All methods will account for correlation of outcomes within clusters and across patients. To test the primary hypotheses, we will use generalized models to assess the effects of the interventions [74]. Our main model will be a mixed-effects (i.e. multilevel) generalized linear model specified as follows: let Y_jkt_ be the average of the j^th^ SPADE score among eligible patients in the k^th^ cluster (*k* = 1, …,21) during step time period t (*t* = 1, …,*T*). In this analysis, weekly time periods (i.e. weekly averages) will be used over 4 years, so that *T* = 208. The SPADE score model is then

$$ {Y}_{jk t}={\mu}_{jt}+{A}_{jk}+{B}_{jk}{X}_{kt}+{\delta}_{jk t}, $$

where

1. *μ*_*jt*_ is a common mean trend for the *j*^*th*^ SPADE score across *t*, shared among all 15 clusters. The multivariate vector ***μ***_*t*_ = [*μ*_1*t*_, …, *μ*_5*t*_]′ is modeled as a multivariate autoregressive process of order 2, AR (2), to allow for more efficient estimation through smoothing of the trend over time.
2. ***A***_*k*_ = [*A*_1*k*_, …, *A*_5*k*_]′ is a vector of random effects (1 for each SPADE score) for cluster k, ***A***_*k*_ ∼ *N*(***ν***_*A*_, ***Σ***_*A*_).
3. *X*_*kt*_ is equal to 0 during the control period for cluster k and is equal to 1 when the intervention begins.
4. ***B***_*k*_ = [*B*_1*k*_, …, *B*_5*k*_]′ is a vector of random effects (1 for each SPADE score) for the effect of the intervention on cluster k, ***B***_*k*_ ∼ *N*(***ν***_*B*_, ***Σ***_*B*_), so that ***ν***_*B*_ = [*ν*_1*B*_, …, *ν*_5*B*_]′ is the average effect that the E2C2 intervention had on the five SPADE scores.
5. ***δ***_*kt*_ = [*δ*_1*kt*_, …, *δ*_5*kt*_]′ are error terms that are treated as a multivariate autoregressive process of order 1, AR (1), with Gaussian noise independently for each cluster.

It can be critical to allow for this correlation in time in longitudinal data [102], which is often ignored in analysis of stepped wedge designs. This primarily results from the complication of fitting the above model, which has several random effects (in both time and across clusters). This is further complicated with five responses due to the multivariate nature of the random effects. However, the Markov chain Monte Carlo method is effective at fitting such models [103] and will be used here. We will assess whether there are any large imbalances in patient characteristics among clusters between the baseline period and intervention period and include any variables wherein large imbalances are detected in the model. The primary aim can be tested with the joint hypotheses that *ν*_1*B*_ = *ν*_2*B*_ = *ν*_3*B*_ = *ν*_4*B*_ = *ν*_5*B*_ = 0. For secondary outcomes, we will estimate a model similar to the main model as well. For all models, we will report measures of fit, such as predictive R^2^ values and between-cluster variance estimates.

A secondary objective of the first aim is to better understand the effectiveness of different components of the E2C2 intervention in managing symptoms, so we will perform a separate set of analyses incorporating only the E2C2 intervention period. For these secondary analyses, we will assess the impact of specific process measures on SPADE scores and other clinical outcomes. We will first examine the distribution of values (frequency or duration) for each process measure and, if the values are highly skewed, we will categorize them into two or more categories. We will then estimate for each outcome a mixed-effects linear model with a random cluster effect, where the outcome is the dependent variable and the main independent variable is the continuous or categorized process measure and adjust for patient covariates as described above. By testing for an overall process measure effect, we can assess which processes contribute most to reducing outcomes; we will also report measures of variance explained to enable relative comparison of process measure effects.

We will use the measures of adoption, fidelity, and penetration (described above) to assess the differential impact of E2C2 components on patient and clinician outcomes. Formally, we will undertake a mediation analysis with structural equation modeling to assess the extent to which each component of E2C2 mediates the intervention.

#### Model estimation

For all models, we will use Bayesian methods to estimate credible intervals for parameters of interest. Bayesian models are appropriate for randomized studies, including cluster randomized stepped wedge studies [104, 105]. Tests will be based on credible intervals for parameters and their combinations.

### Interim analyses {21b}

No interim analyses are planned for this minimal-risk standard of care pragmatic trial. Therefore, no stopping rules have been explicated; analyses will not occur until all sites have been allocated and have completed the planned intervention period.

### Methods for additional analyses (e.g. subgroup analyses) {20b}

For each step, we will first calculate ePRO response rates (the number of ePROs returned divided by the number sent) three times during the pre-E2C2 usual care period (first month, midpoint, and end of pre-E2C2; then again during the first month, midpoint, and end of the E2C2 study period). We will compare rates by age, urban or rural residence, and age or rural residence. To analyze response rate data, we will use both simple unadjusted descriptive statistics and generalized regression models. We will calculate simple proportions of response rates for the total population and subgroups of interest. We will then create regression models to determine predictors of being a high responder. Additional explanatory variables will include patient demographics (e.g. employment status, insurance status, and sex), type and stage of cancer, cluster, and preferred mode of response (ePRO or interactive voice response). Response rates at each time point (early, middle, and late) will be calculated for analyses and models will include a random intercept to account for repeated measures over patients. For each step, we will aggregate data and examine trends over time to determine whether response rates have increased significantly.

We will examine the bivariate relationship between patient, clinician, and utilization characteristics and response with the use of generalized linear regression models testing for interaction effects. Factors that have non-zero interaction effects in this bivariate analysis will be retained; we will use variance decomposition to eliminate collinear variables [106] and enter the remaining factors and their interactions with exposure included in the model. This multistep approach has been used previously in mixed-methods studies to identify the most important predictors [107]. Finally, we will undertake secondary analyses examining the impact of process measures (adoption, fidelity, and penetration) on primary and secondary clinical outcomes among the older and rural subgroups. We expect approximately 20,000–24,000 rurally based patients, 20,000–24,000 elderly patients, and 10,000–12,000 rurally based elderly patients.

### Methods in analysis to handle protocol non-adherence and any statistical methods to handle missing data {20c}

Our approach to data collection, which is designed to ensure complete and balanced collection of primary and secondary measures, is detailed elsewhere (in the “Data collection and management” section). However, we expect that we may be missing some patient or clinician characteristics, which, if otherwise imbalanced across intervention groups, we will need to adjust for; to account for these missing data, we will use multiple imputation, a technique that has been extended to Bayesian models [108].

### Plans to give access to the full protocol, participant level-data, and statistical code {31c}

Given concerns about confidentiality and waiver of consent, datasets generated for the present study will not be made publicly available. Access to the full final protocol, deidentified data, and statistical data will be made available from the study principal investigator upon reasonable request after publication of primary trial results.

## Oversight and monitoring

### Composition of the coordinating center and trial steering committee {5d}

The E2C2 Steering Committee, consisting of the principal investigator, co-investigators, and other study staff, has weekly steering committee meetings to regularly review protocol compliance and to discuss and record study progress.

### Composition of the data monitoring committee, its role, and reporting structure {21a}

The risks posed by the proposed intervention do not exceed the threshold of minimal risk; therefore, an independent data management and monitoring committee is not required. Risks of this study to patients are no different from those encountered during routine clinical care. The principal investigator will monitor the safety of the patients and the integrity of the data. Patient safety will be maintained through the clinical staff adhering to the standards of clinical care.

#### Data integrity

Using best practices in data integrity recommended by the Mayo Clinic Division of Biomedical Statistics and Informatics in the Department of Health Sciences Research, we will use real-time data validation and developed and standardized checklists to ensure data integrity and quality.

### Adverse event reporting and harms {22}

The principal investigator (ALC) will work with the team to develop and submit annual progress reports summarizing study progress to the Mayo Clinic IRB. All unexpected or serious adverse events will be reported by the principal investigator to the IRB. The principal investigator and study team will adhere to IRB policies and procedures related to unanticipated issues involving risk to participants and will immediately report any protocol violations.

### Frequency and plans for auditing trial conduct {23}

This embedded pragmatic trial is being conducted as a minimal-risk standard of care study; therefore, standard operating procedures for auditing trial conduct have not been developed. However, as previously described, we are conducting a rigorous process evaluation involving survey research, focus groups, and key informant interviews, which will provide feedback on trial conduct.

### Plans for communicating important protocol amendments to relevant parties (e.g. trial participants, ethical committees) {25}

All protocol modifications will be submitted as amendments to the approved IRB protocol and communicated to the study team and clinical teams as appropriate.

### Dissemination plans {31a}

#### Potential for impact and implications and plans for dissemination

The E2C2 trial is clinically and methodologically significant because it will comprehensively evaluate the use of ubiquitous EHR functionalities to enhance symptom control. While these capabilities are increasingly EHR agonistic, our use of an Epic system may be particularly beneficial because the systems are used in 250 US healthcare organizations, 65% of the US population has an Epic record, and 69 million Americans have an Epic MyChart account [109]. The proposed trial will have an important impact on the delivery of cancer care, leveraging evidence-based implementation strategies to enhance the adoption, implementation, and sustainability of the intervention in clinical practice. The infrastructural, information technology, economic, and organizational requirements for dissemination on a large scale are integral determinants of the E2C2 approach, which aggressively leverages inexpensive, universally available EHR-based clinical and implementation strategies. Results of the trial will be reported by members of our research team for publication in peer-reviewed scientific journals, and the team will follow authorship requirements as specified in those journals.

During the E2C2 trial, we will develop and refine manuals with specific information for the symptom control– and implementation-focused EHR elements of the E2C2 intervention. These manuals will permit easy dissemination of the approach to builders in other health systems that use the Epic EHR. Epic build manuals will be distributed through several well-established mechanisms, including: (1) Epic online build instruction and support resources; (2) annual Epic Users Group Meetings and online archives of these meetings; and (3) the Cancer Control P.L.A.N.E.T. (plan, link, act, network with evidence-based tools) web platform (https://cancercontrolplanet.cancer.gov/planet/). Since Epic’s market foothold has consistently expanded for the past decade, the potential near-term impact of an Epic-based approach is high.

The E2C2 intervention offers a pragmatic, scalable approach to delivering patient-preferred, guideline-based symptom management that can be easily amended to reflect the dynamic and evolving evidence base. Since discrete EHR-imbedded algorithms drive defining aspects of the intervention, the approach can be efficiently updated by modifying these centralized algorithms. The approach integrates the treatment of psychologic and somatic symptoms in a manner that accords with real-life clinical practice and incorporates treatments spanning different classes—pharmaceutical, rehabilitative, and behavioral. The E2C2 approach can be easily customized to reflect the available services, resources, and organizational structure of specific healthcare systems.

## Discussion

### Limitations and related considerations

#### Administrative challenges

Institutional engagement will be vital for the successful execution of the proposed work and the potential for a lack of engagement inevitably raises concern. We have proactively obtained the support of influential, strategically placed representatives from relevant stakeholder groups throughout MCHS.

The scope of the proposed project requires engagement of a large E2C2 grant team, which may be challenging to coordinate. Diverse stakeholder engagement will be critical for the success of the proposed trial. In planning the grant, we have been strategically inclusive in developing the research and administrative teams.

#### Information technology and clinical workflow challenges

Timely creation of E2C2 clinical decision support tools is also a concern; however, the principal investigator and several members of the study team have been certified by Epic to build and update the E2C2 intervention EHR components.

CAT capabilities are available through all ePRO administration modes: web; smart phone; and tablet. However, CAT administration requires real-time interface with an Epic-based server for item selection. In the event of transient interface compromise, a backup system will administer fixed-length short forms for the domains.

#### Provider challenges

EHR alert fatigue among clinicians has been widely documented in diverse contexts and is an important potential limitation. We will use several strategies to proactively address this concern, including: (1) engaging clinicians in alert design; (2) multi-stakeholder usability testing; (3) site- and cluster-specific vetting of alerts; and (4) qualitative interviews to comprehensively document experiences of end users to improve future iterations.

#### Patient challenges

Access to electronic sources for data collection among patient subgroups is an expected limitation that is central in our mixed methods study design. We will characterize the experiences of patients who may have limited or no information technology access or familiarity, especially rurally dwelling and elderly patients.

Patient engagement is foundational to the success of the proposed system. Patients must actively participate in completion of the ePRO measures and must adhere to recommendations for treatment and self-care for the E2C2 system to be of benefit. We propose to seek input from patients to understand and address barriers to participation and iteratively improve strategies to optimize their engagement.

## Trial status

Ethics approval for this pragmatic trial was obtained on 1 November 2018; the current protocol (version 6) was approved on 16 October 2019. Collection of baseline data (before the intervention) was initiated on 29 March 2019; the intervention was implemented in the clinical practices randomly assigned to tranche 1 on 1 October 2019. The planned field period for this trial extends until 31 January 2023. Thus, the first date of recruitment to the intervention component of this pragmatic trial was 29 March 2019 and the final date of recruitment will be 31 July 2022.

## Supplementary information

**Additional file 1.**

## Data Availability

Given concerns about confidentiality and waiver of consent, datasets generated for the present study will not be made publicly available. Access to the full final protocol, deidentified data, and statistical analysis will be made available from the study principal investigator upon reasonable request after publication of primary trial results. The protocol described herein was registered with ClinicalTrials.gov on 25 March 2019 (No. NCT03892967). Primary findings from this study will be reported to ClinicalTrials.gov.

## Abbreviations

AIM: Acceptability of Intervention Measure
CAT: Computer adaptive test
ED: Emergency department
EHR: Electronic health record
ePRO: Electronic patient-reported outcome
E2C2: Enhanced, electronic health record–facilitated cancer symptom control
FIM: Feasibility of Intervention Measure
IAM: Intervention Appropriateness Measure
IMPACT: Improving the Management of Symptoms During and Following Cancer Treatment
IRB: Institutional Review Board
IRT: Item response theory
MCHS: Mayo Clinic Health System
MCR: Mayo Clinic in Rochester, Minnesota
NCI: National Cancer Institute
NPI: National Provider Identifier
NRS: Numerical rating scale
PROMIS: Patient-Reported Outcomes Measurement Information System
RE-AIM: Reach, Effectiveness, Adoption, Implementation, and Maintenance
RN: Registered nurse
SCM: Symptom care manager
SPADE: Sleep disturbance, pain, anxiety, depression, and energy deficit
