# Supplementary material for: Pragmatic cluster randomized trial to evaluate effectiveness and implementation of enhanced EHR-facilitated cancer symptom control (E2C2)
Source: Trials. 2020 Jun 5;21:480. doi: 10.1186/s13063-020-04335-w (PMC7275300; doi:10.1186/s13063-020-04335-w)
Supplement: Supplementary file 1 — Additional file 1. [file 13063_2020_4335_MOESM1_ESM.doc]

Mayo Clinic: Office for Human Research Protection

**Oral Consent Script—Cancer Care Team Focus Group**

Protocol Title: XXXXXX

IRB #: XXXXX

Principal Investigator: XXXXXXX

If questions or comments, contact: XXXXXX at XXXXXX

You are being asked to participate in a research study about implementing EPIC-facilitated collaborative care for cancer symptoms, sometimes referred to as “E2C2.” You are being invited to participate in a focus group because you play a role in this activity. We want to learn more about you and your team’s experiences related to implementing E2C2.

If you agree to participate you will be asked to participate in a brief, audio-recorded focus group with moderators from the research team. The semi-structured, confidential focus groups will take approximately 60 minutes, and will later be deidentified and transcribed. Audio files will be stored on a secure server. There is no monetary compensation for your participation.

The risks of this research study are minimal, which means that we do not believe that they will be any different than what you would experience during your daily life at work. There may be a small time burden associated with arranging and participating in the focus group. Participants may refuse to answer any and all portions of the focus group.

While there is no direct benefit to you in participating, there is a potential benefit to people in the future as a result of information gathered in the research study.

Please understand your participation is voluntary and you have the right to withdraw your consent or discontinue participation at any time without penalty. Specifically, your current or future medical care or relationship with providers at Mayo Clinic will not be jeopardized if you choose not to participate.

If you have any questions about this research study you can contact [research member] at [phone number]. If you have any concerns, complaints, or general questions about research or your rights as a participant, please contact the Mayo Institutional Review Board (IRB) to speak to someone independent of the research team at 507-266-4000 or toll free at 866-273-4681.
